# (*E*)-*N*-(2,4-Dimeth­oxy­benzyl­idene)-4-ethoxyaniline

**DOI:** 10.1107/S1600536810041759

**Published:** 2010-10-20

**Authors:** Karla Fejfarová, Aliakbar Dehno Khalaji, Michal Dušek

**Affiliations:** a Institute of Physics of the ASCR, v.v.i., Na Slovance 2, 182 21 Praha 8, Czech Republic; b Department of Chemistry, Faculty of Science, Golestan University, Gorgan, Iran; c Institute of Physics, Na Slovance 2, 182 21 Praha 8, Czech Republic

## Abstract

In the title compound, C_17_H_19_NO_3_, the mol­ecule has an *E* configuration with respect to the C=N bond and the dihedral angle between the aromatic rings is 56.07 (5)°. In the crystal, inversion dimers linked by pairs of C—H⋯O hydrogen bonds occur. The dimers are linked by weak C—H⋯π inter­actions, forming a three-dimensional network.

## Related literature

For related structures and background references, see: Khalaji *et al.* (2010*a* ▶,*b* ▶).
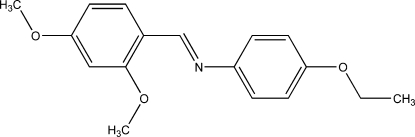


## Experimental

### 

#### Crystal data

C_17_H_19_NO_3_
*M* *_r_* = 285.3
Monoclinic, 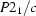

*a* = 8.4536 (1) Å
*b* = 9.6531 (2) Å
*c* = 18.0561 (3) Å
β = 90.9091 (10)°
*V* = 1473.25 (4) Å^3^
*Z* = 4
Cu *K*α radiation
μ = 0.71 mm^−1^
*T* = 120 K
0.50 × 0.12 × 0.11 mm

#### Data collection

Oxford Diffraction Xcalibur diffractometer with Atlas (Gemini ultra Cu) detector
Absorption correction: multi-scan (*CrysAlis PRO*; Oxford Diffraction, 2009 ▶), *T*_min_ = 0.714, *T*_max_ = 1.000
18522 measured reflections
2543 independent reflections
2259 reflections with *I* > 3σ(*I*)
*R*_int_ = 0.024

#### Refinement

*R*[*F* ^2^ > 2σ(*F* ^2^)] = 0.030
*wR*(*F* ^2^) = 0.098
*S* = 1.98
2543 reflections
190 parameters
H-atom parameters constrained
Δρ_max_ = 0.17 e Å^−3^
Δρ_min_ = −0.13 e Å^−3^

### 

Data collection: *CrysAlis PRO* (Oxford Diffraction, 2009 ▶); cell refinement: *CrysAlis PRO*; data reduction: *CrysAlis PRO*; program(s) used to solve structure: *SIR2002* (Burla *et al.*, 2003 ▶); program(s) used to refine structure: *JANA2006* (Petříček *et al.*, 2007 ▶); molecular graphics: *DIAMOND* (Brandenburg & Putz, 2005 ▶); software used to prepare material for publication: *JANA2006*.

## Supplementary Material

Crystal structure: contains datablocks global, I. DOI: 10.1107/S1600536810041759/hb5685sup1.cif

Structure factors: contains datablocks I. DOI: 10.1107/S1600536810041759/hb5685Isup2.hkl

Additional supplementary materials:  crystallographic information; 3D view; checkCIF report

## Figures and Tables

**Table 1:** Hydrogen-bond geometry (Å, °) *Cg*1 and *Cg*2 are the centroids of the C1–C6 and C10–C15 rings, respectively.

*D*—H⋯*A*	*D*—H	H⋯*A*	*D*⋯*A*	*D*—H⋯*A*
C8—H8*c*⋯O2^i^	0.96	2.52	3.3745 (13)	148
C5—H5⋯*Cg*2^ii^	0.96	2.88	3.7529 (11)	152
C14—H14⋯*Cg*1^iii^	0.96	2.76	3.6019 (11)	147

Symmetry codes: (i) 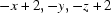; (ii) 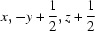; (iii) 
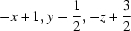
.

**Fig. 1.**
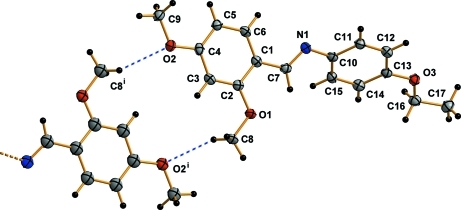
View of (I) with displacement ellipsoids drawn at the 50% probability level. C—H···O bonds are drawn as blue dashed lines. [Symmetry codes: (i) 2 - x, -y, 2 - z]

**Fig. 2.**
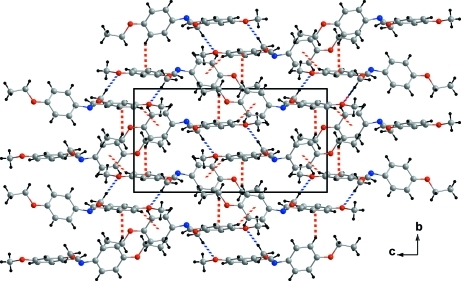
Crystal packing of (I) viewed along the a axis. Hydrogen bonds are displayed as blue dashed lines, C—H···π interactions as red dashed lines.

## supplementary crystallographic information

### Comment

As part of our ongoing studies of Schiff bases (Khalaji *et al.*, 2010*a* and 2010*b*) we now report the synthesis and crystal structure of the title compound, (I).

The title molecule with the atomic numbering scheme is depicted in Fig. 1. The C7—N1 and C10—N1 bond lengths of 1.2795 (13), 1.4171 (13) Å, respectively, conform to the value for a double and single bonds, respectively, and they are similar to the corresponding bond lengths in another Schiff-base compounds (Khalaji *et al.*, 2010*a* and 2010*b*).

The torsion angle of the title compound, C10—N1—C7—C1 - 175.18 (9)°, indicates virtually planar *E*-configuration with respect to the imine C–N bond. Both methoxy groups are nearly coplanar with the C1—C6 ring, as indicated by torsion angles C3—C4—O2—C9 and C1—C2—O1—C8 of 178.16 (9)°, -175.74 (9)°, respectively. The ethoxy group is nearly coplanar with the C10—C15 ring, with the dihedral angle between the ring plane and plane defined by atoms O3, C16 and C17 of 6.28 (9)°.

In the crystal, molecules are connected by weak C—H···O interactions into dimers, which are further linked by C—H···π interactions into a three-dimensional network.

### Refinement

All hydrogen atoms were discernible in difference Fourier maps and could be refined to reasonable geometry. According to common practice H atoms attached to C atoms were nevertheless kept in ideal positions during the refinement. The isotropic atomic displacement parameters of hydrogen atoms were evaluated as 1.2**U*_eq_ of the parent atom.

### Crystal data

**Table:**

C_17_H_19_NO_3_	*F*(000) = 608
*M**_r_* = 285.3	*D*_x_ = 1.286 Mg m^−^^3^
Monoclinic, *P*2_1_/*c*	Cu *K*α radiation, λ = 1.54184 Å
Hall symbol: -P 2ybc	Cell parameters from 13339 reflections
*a* = 8.4536 (1) Å	θ = 4.6–66.4°
*b* = 9.6531 (2) Å	µ = 0.71 mm^−^^1^
*c* = 18.0561 (3) Å	*T* = 120 K
β = 90.9091 (10)°	Prism, colourless
*V* = 1473.25 (4) Å^3^	0.50 × 0.12 × 0.11 mm
*Z* = 4	

### Data collection

**Table:**

Oxford Diffraction Xcalibur diffractometer with Atlas (Gemini ultra Cu) detector	2543 independent reflections
Radiation source: X-ray tube	2259 reflections with *I* > 3σ(*I*)
mirror	*R*_int_ = 0.024
Detector resolution: 10.3784 pixels mm^-1^	θ_max_ = 66.6°, θ_min_ = 4.9°
Rotation method data acquisition using ω scans	*h* = −9→9
Absorption correction: multi-scan (*CrysAlis PRO*; Oxford Diffraction, 2009),	*k* = −11→11
*T*_min_ = 0.714, *T*_max_ = 1.000	*l* = −21→21
18522 measured reflections	

### Refinement

**Table:**

Refinement on *F*^2^	76 constraints
*R*[*F*^2^ > 2σ(*F*^2^)] = 0.030	H-atom parameters constrained
*wR*(*F*^2^) = 0.098	Weighting scheme based on measured s.u.'s *w* = 1/(σ^2^(*I*) + 0.0016*I*^2^)
*S* = 1.98	(Δ/σ)_max_ = 0.013
2543 reflections	Δρ_max_ = 0.17 e Å^−^^3^
190 parameters	Δρ_min_ = −0.13 e Å^−^^3^
0 restraints	

### Special details

**Table:**

Experimental. Empirical absorption correction using spherical harmonics, implemented in SCALE3 ABSPACK scaling algorithm.
Refinement. The refinement was carried out against all reflections. The conventional *R*-factor is always based on *F*. The goodness of fit as well as the weighted *R*-factor are based on *F* and *F*^2^ for refinement carried out on *F* and *F*^2^, respectively. The threshold expression is used only for calculating *R*-factors *etc*. and it is not relevant to the choice of reflections for refinement.The program used for refinement, Jana2006, uses the weighting scheme based on the experimental expectations, see _refine_ls_weighting_details, that does not force *S* to be one. Therefore the values of *S* are usually larger than the ones from the *SHELX* program.

### Fractional atomic coordinates and isotropic or equivalent isotropic displacement parameters (Å^2^)

**Table:**

	*x*	*y*	*z*	*U*_iso_*/*U*_eq_	
O1	0.84529 (8)	0.12294 (8)	0.81770 (4)	0.0248 (2)	
O2	0.78640 (8)	0.15086 (8)	1.07814 (4)	0.0271 (2)	
O3	0.16362 (8)	0.12599 (7)	0.48157 (4)	0.0244 (2)	
N1	0.38690 (10)	0.18316 (9)	0.77253 (5)	0.0231 (3)	
C1	0.59085 (11)	0.15330 (10)	0.86553 (6)	0.0206 (3)	
C2	0.75506 (12)	0.13694 (10)	0.87911 (6)	0.0204 (3)	
C3	0.81587 (11)	0.13725 (10)	0.95058 (6)	0.0220 (3)	
C4	0.71412 (12)	0.15436 (10)	1.00995 (6)	0.0214 (3)	
C5	0.55251 (12)	0.17397 (10)	0.99830 (6)	0.0229 (3)	
C6	0.49391 (12)	0.17310 (10)	0.92638 (6)	0.0222 (3)	
C7	0.52727 (11)	0.14440 (10)	0.79015 (6)	0.0207 (3)	
C8	1.01066 (11)	0.09643 (12)	0.82857 (6)	0.0281 (3)	
C9	0.68947 (13)	0.17194 (12)	1.14125 (6)	0.0284 (3)	
C10	0.33285 (11)	0.16001 (11)	0.69888 (5)	0.0212 (3)	
C11	0.23872 (11)	0.26224 (11)	0.66542 (6)	0.0243 (3)	
C12	0.18457 (11)	0.24746 (11)	0.59337 (6)	0.0240 (3)	
C13	0.22075 (11)	0.12870 (10)	0.55293 (5)	0.0210 (3)	
C14	0.30901 (11)	0.02359 (11)	0.58635 (6)	0.0233 (3)	
C15	0.36467 (11)	0.03995 (11)	0.65886 (6)	0.0229 (3)	
C16	0.19378 (12)	0.00331 (11)	0.43883 (6)	0.0259 (3)	
C17	0.13061 (13)	0.02869 (12)	0.36162 (6)	0.0309 (3)	
H3	0.927413	0.125758	0.959322	0.0265*	
H5	0.483291	0.187796	1.039282	0.0275*	
H6	0.382532	0.186607	0.91805	0.0267*	
H7	0.593525	0.107913	0.752123	0.0248*	
H8a	1.059899	0.085896	0.781357	0.0337*	
H8b	1.05849	0.172598	0.854758	0.0337*	
H8c	1.024488	0.013012	0.856891	0.0337*	
H9a	0.754648	0.173238	1.185267	0.0341*	
H9b	0.634743	0.258688	1.136412	0.0341*	
H9c	0.61388	0.098051	1.144473	0.0341*	
H11	0.211463	0.343686	0.692928	0.0292*	
H12	0.121631	0.319295	0.570909	0.0288*	
H14	0.331465	−0.059847	0.559561	0.028*	
H15	0.425927	−0.032629	0.681647	0.0274*	
H16a	0.139336	−0.07389	0.4602	0.0311*	
H16b	0.305721	−0.012837	0.437197	0.0311*	
H17a	0.142647	−0.053732	0.332495	0.0371*	
H17b	0.020596	0.052809	0.363673	0.0371*	
H17c	0.188299	0.103188	0.339434	0.0371*	

### Atomic displacement parameters (Å^2^)

**Table:**

	*U*^11^	*U*^22^	*U*^33^	*U*^12^	*U*^13^	*U*^23^
O1	0.0173 (4)	0.0359 (4)	0.0213 (4)	0.0025 (3)	0.0015 (3)	−0.0005 (3)
O2	0.0258 (4)	0.0364 (5)	0.0190 (4)	0.0060 (3)	−0.0017 (3)	−0.0013 (3)
O3	0.0267 (4)	0.0261 (4)	0.0204 (4)	0.0031 (3)	−0.0028 (3)	−0.0019 (3)
N1	0.0203 (4)	0.0268 (5)	0.0222 (4)	0.0007 (3)	−0.0013 (3)	0.0002 (3)
C1	0.0193 (5)	0.0176 (5)	0.0248 (5)	−0.0007 (4)	−0.0006 (4)	0.0012 (4)
C2	0.0203 (5)	0.0180 (5)	0.0230 (5)	−0.0002 (4)	0.0021 (4)	0.0004 (4)
C3	0.0183 (5)	0.0225 (5)	0.0252 (6)	0.0013 (4)	−0.0018 (4)	−0.0005 (4)
C4	0.0249 (5)	0.0184 (5)	0.0208 (5)	0.0009 (4)	−0.0026 (4)	0.0001 (4)
C5	0.0233 (5)	0.0230 (5)	0.0226 (5)	0.0016 (4)	0.0038 (4)	0.0002 (4)
C6	0.0172 (5)	0.0224 (5)	0.0271 (5)	0.0009 (4)	−0.0003 (4)	0.0007 (4)
C7	0.0192 (5)	0.0200 (5)	0.0228 (5)	−0.0010 (4)	0.0025 (4)	0.0008 (4)
C8	0.0168 (5)	0.0392 (6)	0.0284 (5)	0.0011 (4)	0.0020 (4)	0.0011 (5)
C9	0.0338 (6)	0.0323 (6)	0.0193 (5)	0.0019 (5)	0.0022 (4)	−0.0003 (4)
C10	0.0155 (5)	0.0261 (5)	0.0222 (5)	−0.0015 (4)	0.0013 (4)	0.0005 (4)
C11	0.0212 (5)	0.0266 (6)	0.0252 (5)	0.0030 (4)	0.0009 (4)	−0.0028 (4)
C12	0.0207 (5)	0.0251 (6)	0.0262 (5)	0.0039 (4)	−0.0008 (4)	0.0010 (4)
C13	0.0174 (5)	0.0250 (5)	0.0206 (5)	−0.0023 (4)	0.0010 (4)	0.0012 (4)
C14	0.0222 (5)	0.0214 (5)	0.0264 (5)	−0.0006 (4)	−0.0002 (4)	−0.0022 (4)
C15	0.0194 (5)	0.0225 (5)	0.0266 (5)	0.0001 (4)	−0.0016 (4)	0.0031 (4)
C16	0.0257 (5)	0.0266 (6)	0.0256 (5)	0.0014 (4)	0.0001 (4)	−0.0040 (4)
C17	0.0347 (6)	0.0332 (6)	0.0248 (5)	0.0006 (5)	−0.0008 (4)	−0.0039 (4)

### Geometric parameters (Å, °)

**Table:**

O1—C2	1.3626 (12)	C8—H8c	0.96
O1—C8	1.4317 (11)	C9—H9a	0.96
O2—C4	1.3662 (12)	C9—H9b	0.96
O2—C9	1.4286 (13)	C9—H9c	0.96
O3—C13	1.3690 (12)	C10—C11	1.3988 (14)
O3—C16	1.4385 (13)	C10—C15	1.3942 (14)
N1—C7	1.2795 (13)	C11—C12	1.3797 (14)
N1—C10	1.4171 (13)	C11—H11	0.96
C1—C2	1.4147 (14)	C12—C13	1.3957 (14)
C1—C6	1.3943 (14)	C12—H12	0.96
C1—C7	1.4578 (14)	C13—C14	1.3915 (14)
C2—C3	1.3814 (14)	C14—C15	1.3932 (14)
C3—C4	1.3948 (14)	C14—H14	0.96
C3—H3	0.96	C15—H15	0.96
C4—C5	1.3919 (14)	C16—C17	1.5051 (15)
C5—C6	1.3824 (14)	C16—H16a	0.96
C5—H5	0.96	C16—H16b	0.96
C6—H6	0.96	C17—H17a	0.96
C7—H7	0.96	C17—H17b	0.96
C8—H8a	0.96	C17—H17c	0.96
C8—H8b	0.96		
			
C2—O1—C8	117.65 (7)	H9a—C9—H9b	109.4721
C4—O2—C9	117.48 (8)	H9a—C9—H9c	109.4713
C13—O3—C16	117.24 (8)	H9b—C9—H9c	109.4713
C7—N1—C10	118.11 (8)	N1—C10—C11	117.83 (9)
C2—C1—C6	117.78 (9)	N1—C10—C15	123.74 (9)
C2—C1—C7	120.11 (9)	C11—C10—C15	118.41 (9)
C6—C1—C7	122.08 (9)	C10—C11—C12	120.81 (10)
O1—C2—C1	115.49 (8)	C10—C11—H11	119.5938
O1—C2—C3	123.75 (9)	C12—C11—H11	119.5935
C1—C2—C3	120.75 (9)	C11—C12—C13	120.40 (9)
C2—C3—C4	119.55 (9)	C11—C12—H12	119.798
C2—C3—H3	120.2241	C13—C12—H12	119.7978
C4—C3—H3	120.2224	O3—C13—C12	115.53 (9)
O2—C4—C3	114.66 (9)	O3—C13—C14	124.96 (9)
O2—C4—C5	124.29 (9)	C12—C13—C14	119.50 (9)
C3—C4—C5	121.05 (9)	C13—C14—C15	119.71 (9)
C4—C5—C6	118.51 (9)	C13—C14—H14	120.1429
C4—C5—H5	120.7434	C15—C14—H14	120.1429
C6—C5—H5	120.7439	C10—C15—C14	121.08 (9)
C1—C6—C5	122.32 (9)	C10—C15—H15	119.46
C1—C6—H6	118.8378	C14—C15—H15	119.4596
C5—C6—H6	118.8386	O3—C16—C17	107.44 (8)
N1—C7—C1	122.80 (9)	O3—C16—H16a	109.4712
N1—C7—H7	118.601	O3—C16—H16b	109.4719
C1—C7—H7	118.601	C17—C16—H16a	109.471
O1—C8—H8a	109.4708	C17—C16—H16b	109.4701
O1—C8—H8b	109.4712	H16a—C16—H16b	111.434
O1—C8—H8c	109.4709	C16—C17—H17a	109.4709
H8a—C8—H8b	109.4718	C16—C17—H17b	109.4708
H8a—C8—H8c	109.4713	C16—C17—H17c	109.4707
H8b—C8—H8c	109.4713	H17a—C17—H17b	109.4715
O2—C9—H9a	109.4707	H17a—C17—H17c	109.4719
O2—C9—H9b	109.4712	H17b—C17—H17c	109.4715
O2—C9—H9c	109.4708		
			
C8—O1—C2—C1	−175.74 (9)	O1—C2—C3—C4	179.02 (9)
C8—O1—C2—C3	4.99 (14)	C1—C2—C3—C4	−0.22 (15)
C9—O2—C4—C3	178.16 (9)	C2—C3—C4—O2	179.02 (9)
C9—O2—C4—C5	−1.49 (14)	C2—C3—C4—C5	−1.32 (15)
C16—O3—C13—C12	−177.94 (8)	O2—C4—C5—C6	−178.96 (9)
C16—O3—C13—C14	1.80 (13)	C3—C4—C5—C6	1.42 (14)
C13—O3—C16—C17	−176.02 (8)	C4—C5—C6—C1	0.03 (16)
C10—N1—C7—C1	−175.18 (9)	N1—C10—C11—C12	178.79 (9)
C7—N1—C10—C11	−141.76 (10)	C15—C10—C11—C12	−2.88 (14)
C7—N1—C10—C15	40.01 (14)	N1—C10—C15—C14	−179.59 (9)
C6—C1—C2—O1	−177.72 (9)	C11—C10—C15—C14	2.20 (14)
C6—C1—C2—C3	1.57 (14)	C10—C11—C12—C13	1.09 (15)
C7—C1—C2—O1	4.41 (13)	C11—C12—C13—O3	−178.79 (9)
C7—C1—C2—C3	−176.30 (9)	C11—C12—C13—C14	1.45 (14)
C2—C1—C6—C5	−1.49 (15)	O3—C13—C14—C15	178.14 (9)
C7—C1—C6—C5	176.34 (9)	C12—C13—C14—C15	−2.12 (14)
C2—C1—C7—N1	−166.67 (10)	C13—C14—C15—C10	0.29 (15)
C6—C1—C7—N1	15.55 (15)		

### Hydrogen-bond geometry (Å, °)

**Table:**

Cg1 and Cg2 are the centroids of the C1–C6 and C10–C15 rings, respectively.

**Table:**

*D*—H···*A*	*D*—H	H···*A*	*D*···*A*	*D*—H···*A*
C8—H8c···O2^i^	0.96	2.52	3.3745 (13)	148
C5—H5···Cg2^ii^	0.96	2.88	3.7529 (11)	152
C14—H14···Cg1^iii^	0.96	2.76	3.6019 (11)	147

Symmetry codes: (i) −*x*+2, −*y*, −*z*+2; (ii) *x*, −*y*+1/2, *z*+1/2; (iii) −*x*+1, *y*−1/2, −*z*+3/2.
